# Clinical Society

**Published:** 1897-12-04

**Authors:** 


### CLINICAL SOCIETY.

### Forcible Straightening of the Spine in Pott's Disease.

The discussion last Friday did not lead to any very definite conclusion. Those who thought that we should hold our hands and await results said so with perfect plainness, while the authors of the papers stuck to their guns, maintaining that the operation had now been done in a very large number of cases; that the accidents had been very few; that even in regard to accidents the treatment compared favourably with other methods which were fully recognised; that in a large number of cases paraplegia, so far from being produced, had been quickly cured; that in many of the cases results were obtained which no other method could show; and that, so far from our holding our hands and waiting to see what happened to the cases operated on in other countries, it was our duty to give our patients the benefit of the operation, and to gain our experience in the method at first hand.

An attempt was made to show that comparable results might be obtained by fixation and steady gradual traction never extending beyond what was comfortable, but the illustrations of the results which had followed these "gentle means" did not seem to carry great conviction to the minds of those present. On the other hand, it was urged that, if the forcible straightening required to be followed by prolonged fixation in an apparatus, that in itself was sufficient to condemn it; the speaker apparently overlooking the fact that this prolonged fixation was exactly the "gentle means" which had been advocated by a previous speaker as the better alternative. It seemed to us that Mr. Spencer sounded the true note when he dealt with the method of forcible straightening not as a mode of treating a deformity resulting from a disease which was past, but as a means of commencing the treatment of a case of Pott's disease in such a manner as to start the child with a straight spine instead of a crooked one. It was not, in his view, a question of curing a deformity, but of starting a child on the right road. The experience which was now at our disposal was at least sufficient to show that, as regards immediate results, there was no danger of making a child worse, and if the anterior common ligament was capable of throwing out ossification, it might as well do it straight as crooked. He thought that, in dealing with early cases, people would now first straighten the spine, and then treat it by fixation.

